# Efficacy of Anlotinib Plus Docetaxel in Advanced NSCLC Previously Treated with Platinum-Based Chemotherapy: A Systematic Review and Meta-Analysis

**DOI:** 10.3390/ph18050652

**Published:** 2025-04-29

**Authors:** Helal F. Hetta, Saleh F. Alqifari, Khaled Alshehri, Amirah Alhowiti, Saud S. Alharbi, Hyder Mirghani, Tariq Alrasheed, Mohamed E. A. Mostafa, Mohammed Sheikh, Mahmoud Elodemi, Sultan A. Alhumaid, Yasmin N. Ramadan, Noura H. Abd Ellah, Reem Sayad

**Affiliations:** 1 Division of Microbiology, Immunology and Biotechnology, Department of Natural Products and Alternative Medicine, Faculty of Pharmacy, University of Tabuk, Tabuk 71491, Saudi Arabia; 2 Department of Pharmacy Practice, Faculty of Pharmacy, University of Tabuk, Tabuk 71491, Saudi Arabia; salqifari@ut.edu.sa; 3 Department of Internal Medicine (Neurology), Faculty of Medicine, University of Tabuk, Tabuk 71491, Saudi Arabia; k_alshehri@ut.edu.sa; 4 Department of Family and Community Medicine, Faculty of Medicine, University of Tabuk, Tabuk 71491, Saudi Arabia; aalhowiti@ut.edu.sa (A.A.); salrehily@ut.edu.sa (S.S.A.); 5 Department of Internal Medicine, Faculty of Medicine, University of Tabuk, Tabuk 71491, Saudi Arabia; h.mirghani@ut.edu.sa (H.M.); talrasheed@ut.edu.sa (T.A.); 6 Department of Anatomy, Faculty of Medicine, University of Tabuk, Tabuk 71491, Saudi Arabia; m_ramdan@ut.edu.sa; 7 Department of Pediatric, Faculty of Medicine, University of Tabuk, Tabuk 71491, Saudi Arabia; msheikh@ut.edu.sa; 8 Department of Pharmacology, Faculty of Medicine, University of Tabuk, Tabuk 71491, Saudi Arabia; malodami@ut.edu.sa; 9 Department of Family Medicine, King Salman Armed Forces Hospital, Tabuk 71491, Saudi Arabia; suaalhumaid@moh.gov.sa; 10 Department of Microbiology and Immunology, Faculty of Pharmacy, Assiut University, Assiut 71515, Egypt; yasmine_mohamed@pharm.aun.edu.eg; 11 Department of Pharmaceutics and Pharmaceutical Technology, Faculty of Pharmacy, Badr University in Assiut, Naser City 2014101, Egypt; nora.1512@aun.edu.eg; 12 Department of Pharmaceutics, Faculty of Pharmacy, Assiut University, Assiut 71515, Egypt; 13 Department of Histology, Faculty of Medicine, Assiut University, Assiut 71515, Egypt; reem.17289806@med.aun.edu.eg

**Keywords:** NSCLC, non-small-cell lung cancer, anlotinib, docetaxel, novel targeted drug, novel multi-targeting tyrosine kinase inhibitor, lung carcinoma

## Abstract

**Background/Objectives**: Anlotinib is a novel oral antiangiogenic tyrosine kinase inhibitor (TKI) approved as a third-line treatment for advanced non-small-cell lung cancer (NSCLC). However, its efficacy in combination with docetaxel remains incompletely understood. Given the need for effective second-line therapies after platinum-based chemotherapy, this systematic review aims to evaluate the therapeutic potential of anlotinib plus docetaxel in advanced NSCLC. **Methods**: The PubMed, WOS, Medline, and Scopus databases were screened for published articles up to 12 April 2024. We included RCTs comparing anlotinib plus docetaxel with docetaxel alone in advanced NSCLC after receiving platinum-based chemotherapy, reporting progression-free survival (PFS), objective response rate (ORR), and disease control rate (DCR) as outcomes for both groups. **Results**: Our systematic review included three randomized controlled trials (RCTs) with a total of 151 patients in the anlotinib plus docetaxel group and 132 in the docetaxel-only group. Meta-analysis results demonstrated that the combination therapy significantly prolonged PFS (mean difference (MD) = 2.98, 95% confidence interval (CI), 1.95–4.00; *p* < 0.00001) and improved ORR (risk ratio (RR) = 3.04, 95% CI = 1.77–5.24; *p* < 0.00001). Additionally, the DCR was notably higher in the combination group (RR = 1.58, 95% CI = 1.34–1.87; *p* < 0.00001). **Conclusions**: Anlotinib plus docetaxel appears to be more effective as a second-line treatment of advanced NSCLC than docetaxel in prolonging PFS and increasing ORR and DCR.

## 1. Introduction

In developed countries, the mortality rate and incidence of lung cancer are the highest. Cancer of the lung is still one of the most common causes of death related to cancer in the USA [1]. It accounts for over 25% of deaths caused by cancer and is the cause of more deaths than the total of colon, breast, and prostate cancer. According to the American Cancer Society, there were around 131,880 lung cancer-related deaths and 235,760 new cases of lung cancer in 2021 [2]. Lung cancer rates are believed to be lower in less developed regions, such as most of Africa and Central/South America. However, many developing countries do not have a centralized reporting system, and many lung cancer cases supposedly go unreported, which hides the true prevalence of the illness [3]. The World Health Organization (WHO) predicts a continuous increase in lung cancer, mostly due to the continuous use of tobacco all over the world, especially in Asia [4]. There are two types of lung cancer: small-cell lung cancer and non-small-cell lung cancer (NSCLC). NSCLC accounts for 85% of lung cancer cases, while small-cell lung cancer (SCLC) accounts for 15% of cases. NSCLC is also divided into three primary types by the WHO: squamous cell carcinoma, adenocarcinoma, and large-cell carcinoma [5,6]. Frequently, the disease progresses to advanced stages before an NSCLC diagnosis is made. The most frequent symptom is cough, which affects 50% to 75% of patients. Chest discomfort, hemoptysis, and dyspnea are the next most prevalent symptoms. Paraneoplastic syndromes and laboratory abnormalities are two less frequent signs. So, a histologic diagnosis of the disease necessitates a biopsy to confirm the diagnosis [7,8]. Determining the tumor’s extent is also necessary for diagnosis. This is accomplished by establishing the TNM stage, which will ultimately determine the available choices for cancer treatment [9,10,11]. The NSCLC treatment is stage-specific. When it is not contraindicated, patients with stage I or II should receive total surgical resection as their course of treatment. Conventional or stereotactic radiation should be an option for nonsurgical individuals. Cryoablation, microwave, and radiofrequency ablation are examples of percutaneous thermal ablation techniques that have been found to be effective treatment choices for palliation in advanced NSCLC and as salvage therapy following surgery, radiation, or chemotherapy [12,13].

Chemotherapy was the mainstay of NSCLC treatment at the start of the twenty-first century. However, a variety of cutting-edge approaches to controlling NSCLC have surfaced since the introduction of immunotherapy [14,15]. Currently, the recommended first-line treatment for patients with metastatic non-small-cell lung cancer (NSCLC) without actionable genomic alterations is immunotherapy, either alone or in combination with platinum-based chemotherapy, depending on PD-L1 expression levels [16,17]. This treatment landscape continues to evolve rapidly, driven by advances in immune checkpoint blockade and biomarker-guided therapies [18,19]. For patients who do not respond to or progress after first-line therapy, treatment options depend on prior exposure to immunotherapy. Furthermore, nivolumab is no longer considered a standard second-line treatment option for patients with metastatic NSCLC, as immunotherapy is now commonly used in the first-line setting based on PD-L1 expression [20]. On the other hand, patients who started with a regimen that included immunotherapy and chemotherapy are switched to monotherapy alternatives as a second-line treatment, such as pemetrexed or docetaxel [16,17]. Unfortunately, docetaxel-based chemotherapy, when used as a second-line treatment, results in inadequate outcomes. It has a median overall survival (OS) of just 5.0 to 8.3 months and progression-free survival (PFS) of only 1.8 to 2.5 months [21,22]. When platinum-based chemotherapy fails to improve survival outcomes, the combination of chemotherapy and antiangiogenic drugs is a potentially promising option. There is a new combination that the oncologists use for the management of advanced NSCLC. A combination of Ramucirumab and docetaxel has been used as a treatment regimen combining chemotherapy and antiangiogenic drugs [23]. Additionally, the LUME-Lung 1 trial—a randomized, double-blind, placebo-controlled Phase III study—evaluated nintedanib in combination with docetaxel as second-line therapy and included a detailed analysis of patient-reported outcomes (PROs). This study highlighted the value of incorporating patient-centered endpoints when assessing treatment benefits, reinforcing the need for a broader understanding of outcomes in this clinical setting [24]. However, more research is needed to determine the safety and proven effectiveness of antiangiogenic drugs as a second-line therapy combined with chemotherapy.

As a third-line treatment for NSCLC, anlotinib is a novel oral antiangiogenic tyrosine kinase inhibitor (TKI) that targets platelet-derived growth factor (PDGF), vascular endothelial growth factor (VEGFR), fibroblast growth factor (FGF), and c-Kit simultaneously [25,26,27]. Anlotinib monotherapy is a third-line strategy that dramatically improves PFS and OS compared to placebo, as the Phase III ALTER0303 trial has shown [26]. Moreover, the combination of anlotinib and chemotherapy has proven to be an effective and tolerable salvage treatment and thus is used for patients with advanced NSCLC as a second- or third-line treatment [28,29]. The precise efficacy of the combination of anlotinib and docetaxel, especially after platinum-based chemotherapy has failed in patients who do not respond well to this treatment, is still not completely understood.

To fill this gap, we performed this meta-analysis to analyze the efficacy of anlotinib and docetaxel combination in patients with advanced NSCLC compared to docetaxel alone.

## 2. Methods

### 2.1. Study Protocol and Registration

We used the Preferred Reporting Items for Systematic Reviews and Meta-Analysis (PRISMA) to conduct this systematic review and meta-analysis. We followed all the steps mentioned in Cochrane’s Handbook of Systematic Reviews of Interventions [30]. This systematic review was registered on PROSPERO: CRD420251033870.

### 2.2. Search Strategy and Data Collection

We searched five electronic databases: Cochrane, Scopus, PubMed, WoS, and Medline via WOS. We searched for all studies published until 12 April 2024. We used the following search strategy: (Anlotinib OR “receptor tyrosine kinase inhibitor” OR “Novel Targeted Drug” OR “novel multi-targeting tyrosine kinase inhibitor”) AND (Carcinoma, Non-Small-Cell Lung OR NSCLC OR Lung Carcinoma, OR Non-small Cell Lung Cancer OR Non-Small-Cell OR Non-Small-Cell Lung Carcinomas OR Lung Carcinomas, Non-Small-Cell OR Carcinoma, Non-Small Cell Lung OR Non-Small-Cell Lung Carcinoma OR Non-Small Cell Lung Carcinoma OR Non-Small Cell Lung Carcinoma OR Non-Small Cell Lung Cancer). We set no limitations regarding study design or year of publication but included only English studies.

We removed duplicates using Endnote Software Version (X-9). We assessed all the retrieved studies for our eligibility criteria in two steps. First, we screened titles and abstracts, and then we screened the full text of the retrieved studies. Studies that met our eligibility criteria were included. Two separate authors performed all the screening steps. Any conflicts were resolved by a third author.

### 2.3. Eligibility Criteria

Studies that met our PICO criteria were included. We included only randomized, controlled trials. The participants of the included studies should have advanced NSCLC who failed to respond to platinum-based chemotherapy to assess the efficacy of anlotinib plus docetaxel compared to docetaxel alone.

### 2.4. Data Extraction and Outcome Measurements

Data were extracted by two separate authors using three Excel sheets, namely summary, baseline, and outcome sheets, and a third author resolved any conflicts. The summary sheet included the study ID, year of publication, study design, setting (country), period of the study, registration ID, regimen, sample size, and type of first-line therapy. The baseline sheet included age (year), male population percent, non-squamous NSCLC percent, and Eastern Cooperative Oncology Group performance status-1 (ECOG PS-1) percent.

The outcome sheet included outcomes used to assess the efficacy of anlotinib and docetaxel: PFS (month), objective response rate (ORR), and disease control rate (DCR).

### 2.5. Definitions of Outcomes

Progression-free survival (PFS): This refers to the time during and after the treatment of the disease when a patient lives with the disease without it worsening. It represents the length of time a patient remains stable without disease progression. For PFS, the interval from the start of the treatment until the detection of disease progression is measured. It does not include patients who die from other causes but focuses on disease stability [31].

Objective response rate (ORR): This is a critical efficacy endpoint; thus, cancer drugs and biologics must be evaluated in terms of this parameter to receive approval. It quantifies the proportion of patients who achieve a specific reduction in tumor size that is sustained over a predefined minimum duration [32,33].

Disease control rate (DCR): This indicates the incidence of participants who respond to the intervention. It provides a holistic perspective on treatment efficacy, considering both tumor shrinkage and disease stability, and is an essential metric in oncology research and patient care [34].

### 2.6. Quality Assessment

As all the included studies were conference abstracts, except one [35], we could not perform a quality assessment of the included studies. No tool has been developed for the quality assessment of conference abstracts.

### 2.7. Data Analysis

We analyzed the data using Review Manager (RevMan) version 5.4. We compared anlotinib plus docetaxel versus docetaxel alone. Continuous data were analyzed as mean difference (MD) using the inverse variance method and a 95% confidence interval (CI). Dichotomous data were analyzed as a risk ratio (RR) using the Mantel–Haenszel method with a 95% CI. A significant difference was defined as a *p*-value of <0.05. Heterogeneity was assessed by forest plot graphs, I-squared (I^2^), and Chi-square (*chi*^2^) tests and was considered significant if I^2^ > 50% and the *p*-value of *chi*^2^ < 0.01. We used a fixed model if the studies were homogenous and a random-effect model if heterogeneity was significant.

## 3. Results

### 3.1. Study Selection

**Figure 1:**
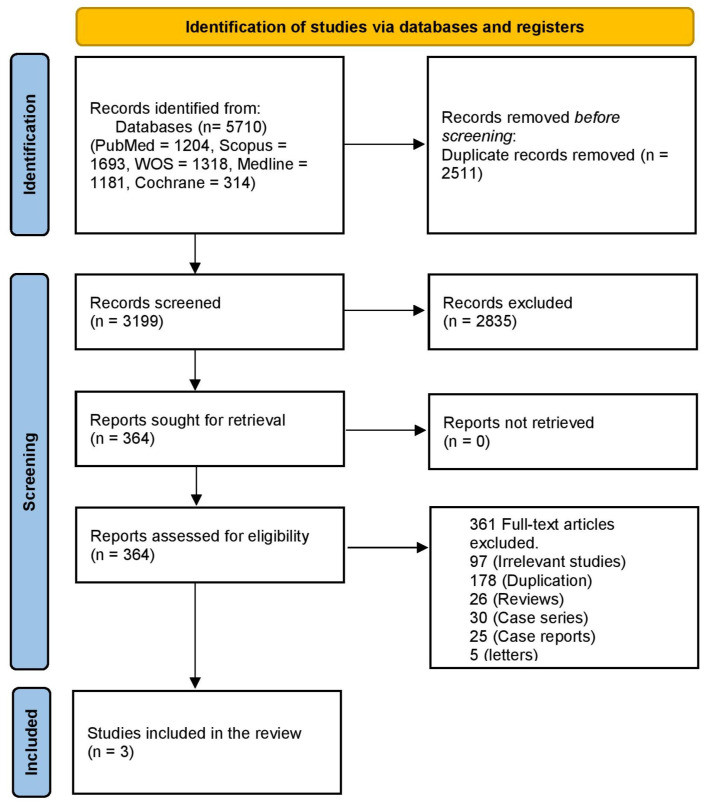
PRISMA flowchart of the study.

A literature search using PubMed, Scopus, WOS, and Medline revealed 5710 articles, 2511 of which were duplicates. Title and abstract screening were performed on 3199 articles, and 364 articles were retrieved. Then, we reviewed them at the full-text screening stage. Finally, 10 studies were included in this systematic review and meta-analysis. The PRISMA flowchart is shown in Figure 1.

### 3.2. Study Characteristics

This systematic review included three studies [35,36,37]. Two of the included studies were conference abstracts of randomized controlled trials that were registered under protocol IDs NCT03624309 and NCT03726736, and one was a completed randomized trial. They were conducted in China. The sample ranged from 40 to 57 in the anlotinib plus docetaxel group and from 31 to 58 in the docetaxel group. The total number of randomized patients was 151 for the docetaxel + anlotinib group and 132 for the docetaxel-only group. The median age ranged from 54 to 62.1 in the anlotinib-plus-docetaxel group and from 58 to 62.9 in the docetaxel-only group. All of the included patients had received platinum-based chemotherapy as a first-line therapy. The summary and baseline characteristics of the included studies are shown in Table 1 and Table 2.

### 3.3. Efficacy Outcomes

#### 3.3.1. Progression-Free Survival (PFS)

**Figure 2:**
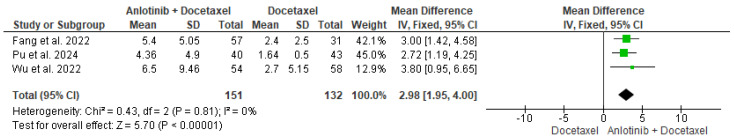
Meta-analysis of progression-free survival [35,36,37].

The data of the three studies, including 151 participants in the intervention group and 132 in the control group, were analyzed and showed a significant difference in the PFS between patients who received anlotinib plus docetaxel and patients who received only docetaxel (MD = 2.98, 95% CI = 1.95–4.00; *p* < 0.00001). The pooled studies were homogenous (*p* = 0.81, I^2^ = 0%) (Figure 2).

#### 3.3.2. Objective Response Rate (ORR)

**Figure 3:**
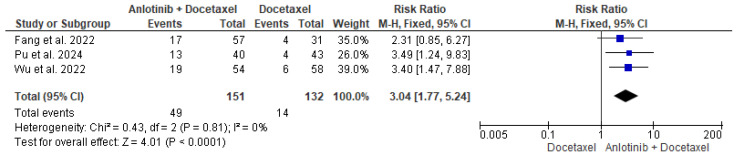
Meta-analysis of objective response rate [35,36,37].

The data of the three studies, including 151 patients in the intervention group and 132 patients in the control group, were analyzed and showed a significant difference in the ORR between patients who received anlotinib and docetaxel and patients who received only docetaxel (RR = 3.04, 95% CI = 1.77–5.24; *p* < 0.00001). The pooled studies were homogenous (*p* = 0.81, I^2^ = 0%) (Figure 3).

#### 3.3.3. Disease Control Rate (DCR)

**Figure 4:**
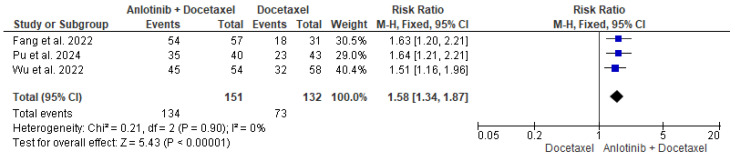
Meta-analysis of disease control rate [35,36,37].

The data of the three studies, including 151 participants in the intervention group and 132 patients in the control group, were analyzed and showed a significant difference in the DCR between patients who received anlotinib and docetaxel and patients who received only docetaxel (RR = 1.58, 95% CI = 1.34–1.87; *p* < 0.00001). The pooled studies were homogenous (*p* = 0.90, I^2^ = 0%) (Figure 4).

## 4. Discussion

In this meta-analysis, we aimed to perform a comprehensive analysis of three RCTs on the efficacy of anlotinib plus docetaxel as a second-line treatment in advanced NSCLC after receiving platinum-based chemotherapy. The results of this study, which report PFS, ORR, and DCR outcomes as primary endpoints, show that the combination of anlotinib plus docetaxel is more effective than docetaxel in several aspects in advanced NSCLC patients.

The analysis showed a significant difference in PFS, ORR, and DCR. The combination of anlotinib and docetaxel increased the mean PFS by 2.98 months compared to docetaxel alone. Although this result is statistically significant, it is considered a modest result in clinical practice. This is due to the short duration of follow-up after receiving the study medication, as mentioned by Pu et al. [35]. So, RCTs with longer follow-up periods are needed to understand the intervention’s effects over time. Our analysis revealed a 29.4% and 91.4% increase in ORR and DCR, respectively (HR = 3.04 and 1.58). These are promising results for clinical practice.

Across the included studies, both treatment regimens—docetaxel alone and docetaxel combined with anlotinib—were associated with a range of treatment-related adverse events (AEs), with notable differences in their frequency and severity [35,36,37].

Patients receiving the combination of docetaxel and anlotinib experienced a higher incidence of grade ≥3 AEs compared to those treated with docetaxel alone. Commonly reported AEs in the combination group included hypertension, hand–foot syndrome, fatigue, diarrhea, and proteinuria, which are consistent with the known toxicity profile of antiangiogenic agents like anlotinib. Hematologic toxicities such as neutropenia and leukopenia were also more frequent in this group, though they were generally manageable with supportive care [35,36,37].

In contrast, the docetaxel monotherapy group primarily experienced chemotherapy-related toxicities such as neutropenia, anemia, nausea, and alopecia, with a lower incidence of non-hematologic AEs.

Despite the increased toxicity observed in the combination group, most studies reported that these adverse events were manageable and did not result in significantly higher treatment discontinuation rates. Overall, the addition of anlotinib to docetaxel was associated with a higher but acceptable toxicity burden, which must be weighed against the observed improvements in PFS, ORR, and DCR [35,36,37,38].

Despite regional variations in immunotherapy accessibility, platinum-based chemotherapy plus immunotherapy has been the accepted first-line treatment for NSCLC in recent years [16,17]. As a result, there has been an increasing interest in investigating more effective second-line therapies, especially ones that use antiangiogenic medicines. Available data indicate that bevacizumab plus docetaxel may be more effective than docetaxel alone in some people [39,40,41]. Furthermore, OS improved from 9.1 months to 10.5 months using the combination of ramucirumab and docetaxel. It also improved the PFS from 3 months to 4.5 months when used as a second-line treatment for patients with stage IV NSCLC [23]. These results are consistent with our findings, which reported a mean PFS of 3.6 months. They are also consistent with the results of an RCT that reported a median PFS of 4.4 months, highlighting the possible advantages of treating patients with advanced NSCLC with chemotherapy and an antiangiogenic TKI [35]. Another clinical trial conducted on non-squamous NSCLC indicated that the docetaxel and apatinib combination was effective and well tolerated [42]. Preclinical research provides evidence that anlotinib can regulate the blood vascular and tumor microenvironments, improving immune cell infiltration and generating beneficial effects when combined with immunotherapy and/or chemotherapy [43,44]. However, there is a need for further research to detect who will have the most benefit from the anlotinib and chemotherapy combination as a second-line treatment.

## 5. Novelty and Strengths of This Study

This is the first meta-analysis performed to assess the efficacy of the anlotinib and docetaxel combination in patients with advanced NSCLC after they failed to respond to platinum-based chemotherapy. It can be concluded that a combined regimen of anlotinib plus docetaxel could be more promising than the standard treatment using docetaxel alone in patients with advanced NSCLC.

## 6. Limitations

Despite these promising results, some limitations must be acknowledged. There is a lack of RCTs that assess the efficacy of anlotinib and docetaxel in advanced NSCLC. Because all of the included studies are conference abstracts, they lack some information about the included patients, such as the percentage of the male population. Additionally, we could not perform a quality assessment of the included studies. The generalizability of our results is limited because all the included studies were conducted in China. Secondly, due to the small population size, the results of this study may be affected to some extent.

One notable limitation of this review is the lack of age-stratified outcomes across the included studies. While age is a critical factor influencing treatment response, tolerability, and prognosis in patients with advanced NSCLC, most of the studies did not report efficacy or safety data according to different age groups. As a result, we were unable to conduct a subgroup analysis to determine whether the benefits or adverse effects of the docetaxel–anlotinib combination varied by age. Future trials should aim to provide age-specific outcomes to better inform treatment decisions for older versus younger patient populations.

Another limitation of our review is the limited and inconsistent reporting of outcomes based on metastatic patterns. While metastatic site and disease burden are known to influence prognosis and treatment response in advanced NSCLC, most of the included studies did not provide detailed stratified data according to the location or extent of metastases. This lack of information prevented a meaningful comparison of treatment efficacy across different metastatic profiles. As such, we acknowledge the need for future studies to investigate how metastatic patterns may modify the therapeutic benefit of docetaxel combined with anlotinib.

An important limitation of the three included studies is that patients had not received first-line immunotherapy with immune checkpoint inhibitors, which are now a cornerstone of treatment for advanced NSCLC without actionable mutations. As such, it remains uncertain whether the observed efficacy of the docetaxel–anlotinib combination would hold in the current clinical context, where most patients receive chemoimmunotherapy regimens in the first-line setting. Widely adopted protocols such as KEYNOTE-189, KEYNOTE-407, CheckMate 9LA, and KEYNOTE-024/042 have significantly altered treatment paradigms and outcomes. Therefore, the applicability of the findings from these earlier studies to today’s patient population may be limited, and further investigation is needed to determine the efficacy of second-line antiangiogenic therapies like anlotinib in patients previously treated with immunotherapy.

It is important to note that the limitations regarding the absence of prior immunotherapy in the included studies also apply to landmark trials such as REVEL and LUME-Lung 1 [23,24]. Both studies were conducted in the pre-chemoimmunotherapy (pre-chemoIO) era, when immune checkpoint inhibitors were not part of standard first-line treatment for advanced NSCLC. Consequently, the applicability of their findings—specifically the use of docetaxel in combination with ramucirumab or nintedanib—as second-line therapy in patients who have progressed on modern chemoIO regimens remains uncertain.

In recent years, several smaller prospective studies have attempted to address this gap. For instance, the VARGADO study evaluated the combination of docetaxel and nintedanib in patients who had previously received immunotherapy, showing encouraging outcomes in this new treatment context [45]. Similarly, the SCORPION study and a prospective observational study of ramucirumab plus docetaxel after combined chemoimmunotherapy have begun to explore the feasibility and effectiveness of this combination post-chemoIO [46]. In addition, multiple retrospective studies have reported real-world experiences with docetaxel and ramucirumab in this setting. These emerging data suggest potential continued relevance for antiangiogenic combinations, but further prospective trials are needed to establish their role in the post-chemoimmunotherapy landscape.

## 7. Recommendations

Future research should involve more randomized controlled trials that assess the efficacy of anlotinib and docetaxel combination in advanced NSCLC as a second-line treatment, especially after the failure of platinum-based chemotherapy. Researchers also should consider performing controlled trials with longer follow-up periods to strengthen the analysis and provide a more comprehensive understanding of the intervention’s effects over time. Future research should also involve a larger sample size to improve the robustness of the results. We also need to conduct studies that focus on adverse drug reactions to provide a clearer understanding of their impact. In any future meta-analysis, a holistic approach should be used for the analysis by considering a combination of studies with longer follow-up periods, larger sample sizes, and clearer insights into adverse drug reactions to mitigate the limitations and enhance the overall quality of the analysis.

## 8. Conclusions

Anlotinib and docetaxel combination appears to be more effective than receiving docetaxel alone for advanced NSCLC. It prolongs PFS and increases ORR and DCR. While the current results provide valuable insights into the effect of anlotinib and docetaxel combination as a second-line treatment in patients with advanced NSCLC, further RCTs are required to obtain more valuable references for the treatment of advanced NSCLC.

## Figures and Tables

**Table 1:** Summary of the included studies.

Study ID	Study Design	Setting (Country)	Date (Period of the Study)	Protocol ID	Anlotinib Plus Docetaxel (Dose/Route of Administration/Num of Cycles)	Docetaxel (Dose/Route of Administration/Number of Cycles)	Sample Size		Type of First-Line Therapy
							Intervention Group	Control Group	
Wu et al., 2022 [36]	Multi-center, randomized, controlled comparative, Phase II trial	China	14 January 2019 and 18 June 2021 (data cutoff: 24 February 2022)	NCT03624309	Anlotinib: 12 mg orally, once daily (QD) from day 1 to 14 of a 21-day cycle; docetaxel: 75 mg/m^2^ intravenously, every 3 weeks (Q3W)	Docetaxel: 75 mg/m^2^ intravenously, every 3 weeks (Q3W); number of cycles not specified	54	58	Platinum-based chemotherapy combined with or without Immune checkpoint inhibitors
Fang et al., 2022 [37]	A Phase II trial multi-center, open-label, randomized controlled trial	China	As of 30 April 2022	NCT03726736	Anlotinib (10 mg, QD, d1 to 14 of a 21-day cycle) plus docetaxel (60 mg/m^2^, Q3W, 4–6 cycles)	Docetaxel (60 mg/m^2^, Q3W, 4–6 cycles)	57	31	Platinum-based chemotherapy
Pu et al., 2024 [35]	A Phase II multicenter, open-label, randomized controlled trial	China	From December 2018 to January 2022	NCT03624309	Oral anlotinib (12 mg/day on days 1–14, +IV docetaxel (75 mg/m^2^ on day 1 of each 3-week cycle)	IV docetaxel at 75 mg/m^2^ on day 1 of every 3-week cycle	40	43	Platinum-based chemotherapy

**Table 2:** Baseline characteristics of patients of the included studies.

Study ID	Groups	Median Age, Years	Male Population *n* (%)	Non-Squamous NSCLC *n* (%)	Eastern Cooperative Oncology Group Performance Status-1 (ECOG PS-1) *n* (%)
Wu et al., 2022 [36]	Anlotinib + Docetaxel	54 (40–71)	33 (82.50%)	25 (55.0%)	27 (67.50%)
	Docetaxel	58 (39–74)	35 (81.40%)	26 (60.47%)	34 (79.07%)
Fang et al., 2022 [37]	Anlotinib + Docetaxel	62.1	NA	32 (65.10%)	46 (80.70%)
	Docetaxel	62.9	NA	12 (73.10%)	26 (71%)
Pu et al., 2024 [35]	Anlotinib + Docetaxel	54 (40–71)	33 (82.50%)	NA	27 (67.50%)
	Docetaxel	58 (39–74)	35 (81.40%)	NA	34 (79.10%)

Abbreviations: NA: not assessed.

## Data Availability

Not applicable.
